# Digital health Systems in Kenyan Public Hospitals: a mixed-methods survey

**DOI:** 10.1186/s12911-019-1005-7

**Published:** 2020-01-06

**Authors:** Naomi Muinga, Steve Magare, Jonathan Monda, Mike English, Hamish Fraser, John Powell, Chris Paton

**Affiliations:** 1 Health services and Research Group, Kenya Medical Research Institute/Wellcome Trust Research Programme, PO Box 43640, Nairobi, 00100 Kenya; 2 Centre for Tropical Medicine and Global Health, Nuffield Department of Medicine, University of Oxford, Oxford, UK; 3 Oman college of Medical Sciences, Muscat, Oman; 4 Nuffield Department of Primary Care Health Sciences, University of Oxford, Oxford, UK; 5 Brown Center for Biomedical Informatics, Brown University, Providence, RI USA

**Keywords:** Digital health, Kenya, Electronic health records, Survey, Health management information systems

## Abstract

**Background:**

As healthcare facilities in Low- and Middle-Income Countries adopt digital health systems to improve hospital administration and patient care, it is important to understand the adoption process and assess the systems’ capabilities. This survey aimed to provide decision-makers with information on the digital health systems landscape and to support the rapidly developing digital health community in Kenya and the region by sharing knowledge.

**Methods:**

We conducted a survey of County Health Records Information Officers (CHRIOs) to determine the extent to which digital health systems in public hospitals that serve as internship training centres in Kenya are adopted. We conducted site visits and interviewed hospital administrators and end users who were at the facility on the day of the visit. We also interviewed digital health system vendors to understand the adoption process from their perspective. Semi-structured interview guides adapted from the literature were used. We identified emergent themes using a thematic analysis from the data.

**Results:**

We obtained information from 39 CHRIOs, 58 hospital managers and system users, and 9 digital health system vendors through semi-structured interviews and completed questionnaires.

From the survey, all facilities mentioned purchased a digital health system primarily for administrative purposes. Radiology and laboratory management systems were commonly standalone systems and there were varying levels of interoperability within facilities that had multiple systems. We only saw one in-patient clinical module in use. Users reported on issues such as system usability, inadequate training, infrastructure and system support. Vendors reported the availability of a wide range of modules, but implementation was constrained by funding, prioritisation of services, users’ lack of confidence in new technologies and lack of appropriate data sharing policies.

**Conclusion:**

Public hospitals in Kenya are increasingly purchasing systems to support administrative functions and this study highlights challenges faced by hospital users and vendors. Significant work is required to ensure interoperability of systems within hospitals and with other government services. Additional studies on clinical usability and the workflow fit of digital health systems are required to ensure efficient system implementation. However, this requires support from key stakeholders including the government, international donors and regional health informatics organisations.

## Introduction

Over the last two decades, digital health systems for hospitals and clinics have been rapidly adopted in high-income countries [1–3]. This move away from paper-based storage and retrieval of medical information to digital systems opens the opportunity for new ways of delivering care and a better understanding of the processes and outcomes of the health service [4–6]. Along the way, these countries have invested large sums and had multiple challenges and failures [3, 7–9]. Interoperability - sharing data between different IT systems and facilities - and usability remain significant barriers to maximising the benefits of digital systems in healthcare [10, 11].

Low-income countries, such as Kenya, are now following this trend and beginning to replace paper-based systems with digital systems [12]. However, the technology landscape is now quite different from 10 years ago, when the systems now in operation in the UK, US and Europe were specified by large national and regional procurement processes [7, 13]. Cloud-hosted systems, mobile phones and tablet computers [14] and increasingly-mainstream adoption of open source technology offer a different and perhaps more cost-effective path for countries looking to digitise their healthcare systems.

Kenya’s healthcare system has recently been devolved, with funding now managed primarily by the 47 counties that make up the country [15]. The central government, through the Ministry of Health, provides support for the counties and, in the area of digital health, has established an eHealth Unit, to guide overall policy, set standards [12, 16] and support national-level systems such as the Master Facilities List (MFL) and the District Health Information Software (DHIS2) (for collating national statistics on health indicators [17]). The eHealth Unit, in conjunction with the Kenya Health Informatics Association (KeHIA), is also leading the implementation of new national projects such as creating a national-level patient identifier, establishing a certification framework for (Health Information Systems) HIS, and working to establish the use of the Digital Health Atlas for ongoing monitoring of HIS implementations in Kenya [18].

In this rapidly changing environment, we investigated the country’s usage of IT in its healthcare facilities and identified trends in the kinds of systems being adopted by public hospitals and the challenges faced. Since the last major survey of systems in 2011 [12], several new central government initiatives have been launched to support IT adoption in hospitals and new IT vendors and non-governmental organisations have updated existing systems and created new products for the Kenyan healthcare market. There is also limited literature on the implementation of EHR systems at the hospital level that is not tied to a specific disease such as HIV.

This survey aims to provide decision-makers with information on the digital health systems landscape and to support the rapidly developing digital health community in Kenya and the wider region to share knowledge and experience. We do this by identifying digital health systems implemented in hospitals and gathering views about system implementations from users and vendors. In addition to the results presented here, we also conducted and published a separate case study of an EHR implementation to drill down into the process of implementing IT systems in low-resource settings such as Kenya [19].

## Methods

### Survey

We surveyed County Health Records Information Officers (CHRIOs) to determine the levels of IT adoption in public hospitals across Kenya. HRIOs are the custodians of health information with their roles spanning data collation and reporting at the aggregate level. The survey was conducted at a National County HRIO meeting organised by the Ministry of Health. We took advantage of this gathering and requested all participants to fill out a paper survey. Email and telephone follow up was used to clarify arising issues. The survey asked HRIOs to list the IT systems used in facilities within their counties across the major hospital functions: patient registration, billing, outpatient, in-patient, pharmacy, laboratory, finance, human resources (HR), and comprehensive care clinics (outpatient clinics for HIV care and treatment services also referred to as CCC). Our focus was on public hospitals that serve as internship training centres in Kenya (level 4–6). Internship training centres receive high volumes of patients and are likely to have implemented an electronic system to facilitate service provision to their clients.

Health service delivery in Kenya is a devolved function run by 47 counties. The health delivery system is currently classified into six levels of care, expected to transition into 4 levels, with different facilities falling into the levels according to the services they provide [20] as summarized in Table 1.

**Table 1:** Organisation of the Kenyan Health system - adapted from Kenya Health Policy 2014–2030

Current levels of care	Desired Levels of care	Facilities	Description
Level 1: Community	Level 1: Community	The village, households, families, individuals	• Community-based health services
Level 2: Dispensaries and clinics Level 3: Health centres	Level 2: Primary care facilities	Dispensaries, clinics and Health centres, maternity homes	• Disease prevention and health promotion services • Inpatient services for emergency clients awaiting referral, clients for observation, and normal delivery services
Level 4: Primary care hospitals Level 5: Secondary care hospitals	Level 3: County hospitals	Primary care hospitals Secondary care hospitals	• Comprehensive inpatient diagnostic, medical, surgical and rehabilitative care, including reproductive health services • Specialised outpatient services • Hospitals managed by a county
Level 6: Tertiary care hospitals	Level 4: National referral hospitals	Tertiary care hospitals	• Tertiary/highly specialised services, including high-level specialist medical care, reference laboratory support, blood transfusion services, and research • have defined level of self-autonomy

### Digital health system identification and interviews

Through a snowballing process, we aimed to identify vendors supplying digital health systems to public hospitals in the country. An initial list of 17 digital health systems was retrieved from a 2011 review report by the Ministry of Health [21]. A web-based search and further consultations with the Kenya Health Informatics Association (KeHIA), Ministry of Health staff and other stakeholders broadly involved with digital health in the country was performed to produce a more up-to-date list of systems. We aimed to identify major systems used for administrative functions as well as clinical data collection in large hospitals that serve as internship training centres in Kenya.

To develop the interview guides, we searched the literature for similar work, frameworks and models that have been used in evaluating digital health implementations [16, 22–24]. Additionally, guidelines for reporting evaluation of studies in health informatics [25] were considered to create a semi-structured interview guide comprising both open- and close-ended questions. The comprehensive questionnaire was split into a section for hospital users and vendors. It was piloted at two facilities then revised to shorten and refine it for clarity.

For each major system identified, we aimed to conduct at least one site visit to a facility where the system was in use to conduct semi-structured interviews with stakeholders including the hospital management, IT staff and clinical end-users who were knowledgeable about their institution’s digital health system adoption and were available on the day of the visit.

Where systems were widely used, we attempted to conduct face-to-face interviews with the system vendors and view demonstrations of their systems where possible to get their views on system implementation. We obtained vendor contacts where possible and sent the questionnaire ahead of time. We followed this up with interviews to clarify issues arising from facility visits or the questionnaire that was sent to them. During the interviews, two members of the team were available to take notes and conduct the interview.

Data were collected between April 2014 and November 2016. Both the survey and the interviews were conducted in English. One researcher read all the transcribed interview transcripts and through an iterative process of re-reading and coding, identified emergent themes using a thematic analysis from the data using QDA Miner Lite 2.0.5 software.

### Ethics

Ethical approval for this study was granted by the Kenya Medical Research Institute (KEMRI) Scientific and Ethics Review Unit (SSC protocol No. 3166). We obtained written consent to take part in the study from the participants prior to the start of the interviews. An information sheet giving a summary of the study purpose and procedures and outlining that participation was voluntary and that they were free to withdraw at any stage was attached to the consent sheet.

## Results

We obtained information from 39 CHRIOs, 58 hospital managers and system users across various departments of 13 hospitals where EHRs were implemented through semi-structured interviews and filled questionnaires and 9 system vendors who responded to the questionnaire and agreed to an interview. The number of participants in the hospital visits ranged from 1 to 10.

### Survey results

A total of 39 CHRIOs responded to the survey and gave information on 121 public health facilities (level 4 to level 6) with each CHRIO representing a county. The CCC department was the most computerised (88%, *N* = 121) followed by outpatient (38%, N = 121) and billing (26% N = 121). One system vendor had an inpatient module available for clinical care where the clinical users could access paediatric inpatient data while the rest only provided ward management modules for inpatient services such as: ward search, bed allocation, transfers and bill management.

Hospitals were commonly found to have different software in the CCC to other departments. Table 2 shows a summary of computerized departments for level 4–6 facilities. Most of the systems encountered were open source software (11/18) serving the CCC as part of wider donor funded efforts to support HIV care. There were 7 proprietary systems serving the other departments within the hospitals that were purchased by the hospitals. Our snowballing process revealed that there were other systems that were used in the private hospitals but that were not in use in the public hospitals we surveyed.

**Table 2:** Summary of computerised departments

Department (number of facilities = 121)									
CCC	Inpatient	Outpatient	Pharmacy	Laboratory	Billing	TB	Maternity	MCH	Radiology
106 (88%)	8 (7%)	46 (38%)	28 (23%)	28 (23%)	31 (26%)	8 (7%)	5 (4%)	10 (8%)	8 (7%)

Table 3 presents a summary of system characteristics for the systems identified

**Table 3:** Digital health systems characteristics

	Proprietary	Open source	Coding	data exchange	Common Modules
Hospital Management Systems	*n* = 7	*n* = 0	ICD10	HL7 XML DICOM SQL CSV	Registration, billing, outpatient-clinical, pharmacy, laboratory, finance, Human resources, ^a^inpatient-administration
CCC systems	n = 0	*n* = 11	• CIEL/MVP concept dictionary with reference to ICD10 and SNOMED-CT • CPT4 • RxNorm	HL7 REST API XML	Registration, laboratory and pharmacy results,

^a^ inpatient-administration: ward search, bed allocation, transfers and bill management

### Interview findings

A total of 58 participants gave their views either by participating in semi-structured interviews or completing the semi-structured interview guide section on system views. The participants included hospital managers, clinical staff in the outpatient department, laboratory, pharmacy, ICT support as well as health records and information officers drawn from departments where digital health systems were implemented. We elicited information on the history of system acquisition and implementation, system support, data use and general views on system use. Six themes emerged from the hospital interviews and five from the vendor interviews from the thematic analysis of the interview findings. The key themes are summarised below, with illustrative quotes. A summary chart of the coding for both hospital and vendor interviews is provided in Appendix.

### Hospital interview findings

#### System acquisition

Hospital managers often cited the need for financial accountability as a primary reason for purchasing EHR systems. Other reasons for implementing a digital health system included: to improve service delivery, challenges with previous systems, previous experience (of managers) with a digital health system in another facility and a need to manage clinical data. The systems were sourced by the hospital and in some cases, sourced at the county level through a tendering process or recommendation by the Ministry of Health through direct purchase using hospital funds. In some instances, where pilot systems were in place, the system was donated to the facility. Integration of the outpatient billing module with accounting and finance modules was considered an essential feature in system selection.

*Manager H13: “And we used it mainly for collecting revenue, but eventually it was developed to take care of the other services, as the main-focus was the out-patient, where we were able to withdraw all the papers and we were able to use the computers to offer the services, [such] as clerking of the patients.”*

Monitoring and accountability were reported to have improved in different departments and this was appreciated by the health workers but most especially where revenue collection was concerned. Other benefits included: increased revenue, invoices reprints, controlled transaction reversal, payment tracking, patient scheduling and stock levels monitoring.

#### System support, acceptance and user training

Most of the hospitals surveyed had an ICT department which addressed basic user issues and performed advanced EHR support to a limited extent with support from the system vendor. In some cases, health records information officers provided IT system support services in addition to their own tasks. The ICT departments’ responsibilities also included adding and training new users, assigning to them appropriate roles and performing system backups. Infrastructure issues such as breakdown of IT equipment and network connectivity problems were usually resolved within a relatively short period.

With regards to vendor support, system users reported varied response times to proposed change requests because of the prioritisation of emerging issues. Despite hospitals often having ongoing support contracts, request for changes fell out of scope for support. In some facilities, management cited long procedures for procurement of services and budgetary constraints as a major cause of delays in getting IT service providers to act on queries.

*Hospital manager H4: “Mostly immediate but depends on the priority of the problem/where in the hospital the problem has occurred. Problems at billing and registration are dealt with a lot sooner than other areas.”*

Initial system resistance and computer illiteracy among staff are some of the challenges encountered during system implementation phase while some users would look for shortcuts or workarounds due to unfamiliarity with the system. In some facilities, the system became more acceptable after a period of use and user training on both computer and system use.

#### Usability

System users generally reported that the systems were fast, made their work easier, were easy to use and user friendly. This was attributed to faster data entry once the users were well trained and acquainted with the system.

However, system usability was affected by issues such as lack of integration with other systems both within and outside the facility, lack of computerisation in other departments, system workflow issues, poor uptake by other system users and software crashes. It was also mentioned that there were instances where physical forms were required as part of government regulations making it difficult to use available system features; this was in relation to the financial information management module.

*ICT Admin H4: “Government forms that require original hardcopy forms make certain reports from the system unusable. Manual copies have to be filled out, stamped etc for physical delivery to relevant government offices”.*

#### Departmental communication and system interoperability

There were varying levels of interoperability between systems within the same facility. In some instances, systems were integrated and able to exchange data (e.g. laboratory system connected to equipment enabling automatic transmission of results) while in another case, one department had access to two systems that were not interoperable. This created a challenge in ensuring all the relevant data were captured; the vendors were in discussion to implement interoperability. Some users mentioned that the use of electronic systems made it easier to communicate with health workers in other departments.

*System user H11:” No, we don’t currently [re-enter data] we are not putting them back to the [system] neither are we putting it back to the [other system] because of those challenges. We get the information from the patient, you’ll have to have some extra time or another person doing the feeding [of data] to the other system, this is a challenge to us because of the second part [entering the data again] …. Already we raised the issues so the vendor for [system] and the vendor for [other system] are trying to work on that so that they can communicate”*

#### Report generation and data issues

Health workers reported having more access to data and could generate some reports required by the facility and the Ministry of Health. Auditing of previous prescriptions, easier file retrieval and ability to access records for later reference were mentioned as benefits of the systems. In addition, prescription errors were reported to have reduced, dispensing made easier and automation of work led to less paper work.

However, non-entry of clinical data by nurses and clinicians was reported to be a persistent issue and this affected the generation of certain reports. There was a mismatch between diagnosis offered in the system and what the clinicians wanted to enter for their patients while in some cases, some reports were still not available from the systems.

*Hospital manager H6: “Diagnoses offered by the system sometimes don’t match with the clinician’s impressions. And they may sometimes just click anything close so that they can move onto the next item required by the system.”*

For pharmacy and administrative users, system benefits included: informed procurement decisions, decreased wastage and reduced costs. Administrative users in the pharmacy, finance and accounting departments noted that the cohesive nature of the modules within the system reduced the time spent in reconciling information and preparation of reports. Increased patient confidentiality was also seen as a benefit as access to patient information was restricted.

#### Infrastructure

Infrastructure challenges were identified as some of the barriers to effective digital health system use in all the facilities. These included inadequate numbers of computers, missing computer peripherals and damaged network equipment. Theft of equipment also emerged as a barrier to system use. To overcome the issue of theft, one facility required staff members to take personal responsibility for the loss of equipment. To mitigate some of these challenges, some facilities ensured local IT support was available to ensure quick response to equipment breakdown and allocated more funds for system support.

Electrical power interruptions were also a challenge to system implementation. In some cases, parts of the hospital were connected to a generator while some departments were not. In some facilities, when there was no power supply, there were no clear guidelines on an alternative means of data entry which resulted in long patient queues.

*ICT manager H4: “No work around during system downtime during power black outs; patients have to wait.”*

### Vendor technical interviews

We interviewed 9 system vendors who were Kenyan software development companies that sold commercial digital health solutions (although many using an open-source technical platform or “stack” developed locally) to public or faith-based facilities.

#### Data and reporting

Various data coding terminologies were included in the digital health systems encountered in this survey. The International Statistical Classification of Diseases and Related Health Problems 10th Revision (ICD10) classification was used for diagnosis. SNOMED CT (Systematized Nomenclature of Medicine -- Clinical Terms), Digital Imaging and Communications in Medicine (DICOM) and Current Procedural Terminology (CPT4) standards were also reported to be in use. HL7 was also available to facilitate data transmission between systems but we did not observe this. However, it was reported that some users would request free text fields rather than use standardised coding during data entry.

The lack of a national unique identifier was a challenge that was voiced by the system vendors. Facilities had different identifiers for different departments and this was cited as a challenge in implementing a digital health system effectively.

*Vendor 8: “But when you go and try to give that [unique patient identifiers] to the government facility you find there are some people who are against even changing the MCH [Maternal and Child Health] numbers so you find that normally when it is manual, they have the have the ANC [Antenatal Care – for mothers] number they have the CWC [Child Welfare Clinic] for kids. But the same kid will be treated now as an outpatient will be given another number. So, we are telling them when it comes to electronics normally, the only way you can control this person is by using one number.”*

The vendors reported the ability to generate both local reports and reports required by the Ministry of Health such as MOH 705A (Out Patient Summary Sheet Under 5 Years), 705B (Out Patient Summary Sheet Over 5 Years) and 718 (Inpatient Morbidity and Mortality Summary Sheet).

#### Support to facilities

The system vendors provided various levels of support before, during, and after the implementation period. The systems were often customised to meet the requirements of the hospitals and reporting guidelines set by the Ministry of Health. This support was provided in accordance with the available maintenance contract, subject to availability of funds and priority of the issue that required support.

*Vendor 5: “But of course, you know sometimes some of them are really urgent. They just call us that there is a patient with a doctor, their medical report is not opening so how do we go about it. Either via TeamViewer or whatever else, I can log in and be able to sort it out.”*

Vendors worked with each other and with trusted IT staff within the facilities to carry out minor changes to the software for non-sensitive modification. In one case, one vendor worked closely with another to provide support because of a previous working relationship implying that there was some trust between the vendors that had been cultivated. The IT staff allowed to make changes were identified by the hospital managers. Remote support tools such as TeamViewer and Skype were used to provide support for issues that could be resolved remotely. The support given to facilities was documented through WhatsApp or email communication to hospital administrators or through a dedicated system supported by service request specification documents.

#### User related challenges

Vendors faced various user-related challenges during system implementation such as a perceived negative attitude towards the implementation of electronic systems and computer illiteracy. The county governments were reported to be instrumental in dealing with this by issuing directives that county hospitals had to enforce the use of digital health systems.

*Vendor 8: “With the counties, because monitoring is near to the facilities these guys [system users] now are forced to work. Before, when health used to be managed from AFYA house[building where the Ministry of Health is located in Nairobi, Kenya], by the time you go to [location Y] it may take months, so these guys [system users] used to relax but nowadays once a project is set in place, the infrastructure is there, the staffing is done properly, you are given an option, you either use the system or look for an alternative.”*

To overcome the user-related challenges, some vendors trained the users and provided technical and user documentation (written and video form) as part of their key deliverables, to enable the users to become proficient in both digital health systems and general computer use.

#### System related

Some vendors reported offering comprehensive system solutions that cover all departments should a facility wish to implement all modules. However, there were different levels of implementation of one system in different facilities depending on the facility’s needs, size and purchasing power. Some vendors mentioned that they had plans to implement a patient portal. Additionally, inadequate supply of required hardware and infrastructure hindered facilities from implementing all available modules. System implementation was found to be prioritised on cash collection points and once those were well done then management commissioned deployment to clinical areas.

*Vendor 2: “There is no facility which is using all of them[modules], but for those who have ordered the full system, now they start with the most critical. You know you can do them in modules then they start like this: revenue, registration of patients and admissions then they go to these clinical areas, supply chain because they want to monitor commodities and others go to HR payroll and all that “*

In cases where the facility already had an electronic system within a department, efforts were made by the vendor to ensure mechanisms of exchange data were either in place or were work in progress. The HL7 standard to enable transmission of data across systems was reported to be supported by some vendors in the survey although we did not observe the use of HL7 for interoperability during facility site visits. One vendor indicated that integration with other hospital equipment in the laboratory and imaging departments was a priority. Methods used to share data between systems included: XML, CSV files for data transfer or direct system integration at the database level.

Changes to data collection templates from MOH was mentioned as a challenge to implementing digital health systems as it necessitated changes to system templates, often on short notice. However, the vendors appreciated that once a form was done for one facility the update would be shared throughout other implementations. The vendors also customised reports that were required by the Ministry of Health for aggregate data reporting via DHIS2. These reports would then be downloaded and manually entered into the DHIS2 system. Vendors reported willingness to integrate directly with DHIS2 but were unable to implement this feature due to a lack of a framework that specifies how digital health systems can integrate with the national data aggregation system.

Data was backed up by implementing either redundancy to ensure system availability in case of a failure or through scheduled automatic, encrypted data dumps. The backup location varied depending on the facility and what was deemed as acceptable. Additionally, vendors offered off-site backup, but the users were concerned about the safety of the data given its sensitive nature in addition to cost implications, especially where large files were involved, that many facilities would not be able to bear. Cloud-based servers for smaller facilities that were not able to hire support staff was an option available to enable easier management of software.

#### Legislation, governance and national programs

The county directive to install county-wide and interconnected systems was met with challenges of inadequate infrastructure. County governments also initiated change of already implemented systems to newer ones but in some cases, this did not work, and hospitals had to revert to the previous system. Change in systems also meant loss of previously available reports.

*Vendor 8: “They have started, what has happened with [system X], they are piloting in [hospital]. Because you see [system X] was from a private [facility] now they are trying to customize into the government way of working. Now it is becoming a bit of a challenge to have and you see these reports take time. I know eventually they will do it, but you see they have been getting some reports and now they not getting them.”*

In one county, there were plans to implement a wide area network to facilitate patient follow up and motivated by the county’s need to minimise wastage. For example, patients were reported to collect drugs from several health facilities where services are provided free of charge but there was no means by which facility B could verify that a patient had already collected the drugs from facility A.

Other challenges that affected digital health system implementation were related to national country programs such as the National Health Insurance Fund (NHIF). The NHIF has programs for different services provided to its members. This then affected the way the vendors could implement certain features within the systems.

## Discussion

### Principal results

In this study, we found that public hospitals commonly purchased systems for patient administration and hospital billing functions and the same systems were also used to manage the provision of clinical services in the outpatient department. The radiology and laboratory management systems were commonly standalone system with varying levels of interoperability. From the user perspective, issues such as system usability, adequate training, availability of adequate infrastructure and system support emerged. The vendors were found to have a wide range of modules available on their systems, but their implementation was constrained by limited hospital funding, lack of policies to facilitate features such as data sharing and lack of users’ confidence in new technologies.

In the hospitals we visited (and supported by literature [12, 16, 21, 26, 27]), there appear to be two parallel patterns of digital health system implementation in public hospitals in Kenya: a top-down approach fostered by the MoH in partnership with development organisations that primarily use open source systems such as OpenMRS; and a bottom-up approach implemented by several smaller hospitals and using locally developed commercial systems (although many using an open-source technical platform or “stack”).

“Top-down” systems such as the donor supported open source systems primarily used in providing care to HIV patients have been found to be highly compliant to the standards and guidelines for EMRs set for Kenya [12]. These systems have the potential to be extended to other clinical areas and possibly modified for administrative functions. The Afya-EHMS project [19], Banda Health (both modifications of the OpenMRS system) and IQCare, primarily used in HIV care, are examples of HIV systems being customized to extend their functionality.

In a “bottom-up” approach, public hospitals in this survey also mobilized funds to procure proprietary software to meet their administrative needs and attempted to extend these systems to clinical care amidst challenges of inadequate funding, infrastructure and human resource capacity. This implies that more established administrative systems also have the potential to be extended to clinical areas and, where possible, interface with existing systems if adequately funded and supported. This also requires that systems adhere to standards (both local and international) in digital health system development and implementation if the interoperability and integration is to be successful.

Workflow issues may arise if workflow evaluations are not conducted prior to system implementations. Mapping current workflow processes - both within the hospital and the wider health ecosystem - and evaluating how digital health systems fit in are a critical step in system implementation often leading to re-organising work processes in order to reduce inefficiencies or remove duplicative processes [28]. These workflow issues, in turn, can influence negatively the usability of the system and subsequently quality of data generated from a system making it unsuitable for use. Poor documentation of clinical data was identified in this study as a barrier to generation of usable reports that were required by the Ministry of Health. Poor clinical data was cited due to health workers’ lack of time to enter data, mismatch between system and clinician diagnosis, and systems that were not interoperable.

The ability to exchange data between systems emerged as an issue both at the hospital and vendor level. Because some systems were not integrated, some users at health facilities had to use two independent systems within a department. At the aggregate level, users were required to generate reports at the facility and then have the data entered manually into the national health information system. At the technical level, interoperability was implemented variably or was a work in progress at the time of the survey. However, it appears that there are wider managerial issues at the hospital level and data exchange framework issues at both county and national level that need to be addressed to facilitate seamless data exchange. Seamless data exchange is an important foundation for achieving integrated digital health systems that support long term patient-centric care that is provided across different sites. Senior management attitude towards the HL7 interoperability standard, staff’s technology capability, system integrity, and hospital’s scale are critical factors influencing hospitals’ intention on whether to adopt HL7 [10].

We observed laboratory systems that were provided by a separate vendor from the administrative system used to manage the laboratory services. In one facility, the lab system was reported to be interoperable with other systems within the facility while in other facilities, lab results had to be manually entered onto the administrative system.

The end users of the systems implemented in the hospitals we visited were, in general, happy to use the systems provided it met their needs and the reporting needs of the Ministry of Health. However, they are faced with challenges which, if not addressed, could lead to frustrations and rejection of the systems. Power supply issues are common in developing countries [6, 29–31] and have been addressed by a redundant power supply to areas where digital health systems are in use. The use of Uninterrupted Power Supply units (UPS) and alternative power sources such as accumulators and inverters [29] or deep-cycle batteries used for solar power installations [31] have been used in Malawi to supply electricity for extended periods (36–48 h) during power blackouts. Some users also felt that the training and system support provided by the system vendors was still inadequate despite receiving training on general computer and system use. Adequate support provision was also constrained by the availability of funds.

The system vendors were willing to support newer technologies such as cloud-based systems and provide additional modules to the hospital such as patient portals and inpatient modules for clinical data entry. Users were, however, concerned about privacy issues owing to the nature of data collected within a hospital and the cost implications. Cloud-based systems may offer respite for small facilities that cannot afford to install and maintain sophisticated hardware at a facility. They do, however, require good internet connectivity or at least a hybrid model that stores data locally and then synchronises whenever there is a stable internet connection [32]. This then opens possibilities of offering seamless patient care across facilities.

In a survey of health workers in Kenyan public hospitals, the need to access information on their own performance of routine clinical tasks (care given to patients) was found to be important to health workers but hospitals are not able to provide this information [33]. This ability to meet the health worker’s need is further confounded by constraints such as data quality assurance, data infrastructure with respect to the information and communications technology applications, human resources, financial resources, and integration (linking local digital health systems and DHIS2) [34]. The current digital health system implementations, if carefully implemented, could begin to bridge these gaps and help hospitals meet the information needs (feedback on care provided to patients) of their health workers and those of the national health information systems.

Digital health systems that support the collection of standardized data as part of routine care across multiple facilities are just emerging in many LMIC provide a digital infrastructure to support learning health systems. The Institute of Medicine views digital health systems as an essential part of a learning health care system or a system that is “designed to both generate and apply evidence to promote innovation, quality, safety and efficiency in health care” [35]. Emerging work on learning health systems shows that with the appropriate structures in place such as research and capacity building, governance, infrastructure and funding, developing countries can strengthen routinely collected data to conduct pragmatic, contextually appropriate research and rapid adoption of findings to provide quality care [36]. Our findings reveal that there are opportunities to strengthen digital health systems in the area of appropriate infrastructure (policy, physical and human resource) towards building learning health systems in Kenya to realise the full benefits of digitisation of health records.

### Limitations

There was some difficulty in confirming systems in use owing to a lack of response to follow up emails and calls. The survey required the County HRIOs to list systems within their county and which facilities were using these systems. These results may have been affected by recall bias. One facility that was visited earlier in the study was found to have been engaged in new discussions to implement a different system than the one that was previously piloted while others were in the process of procuring new systems, making difficult to follow up on the progress of the piloted system. The survey responses from the qualitative interviews may not be reflective of all issues that exist with the system implementations as people might have felt then need to report the positive aspects of system implementation and downplaying the existing challenges (desirability bias).

### Future work

Our work aimed to produce a general view of digital health systems implementations in Kenya from a variety of hospitals. This work has revealed a range of issues that would need to be explored or studied to give a better understanding of the digital health landscape. Such issues include a detailed description of available infrastructure, an assessment of staff numbers and their ability to analyse data, a better understanding of procurement processes for digital health systems, an enquiry as to how these digital health systems fit in with the wider ecosystem such as financial information management and policy implications in related sectors.

## Conclusion

This study highlights the challenges faced by both public hospital administrators and vendors in implementing digital health systems in developing countries such as Kenya. These challenges provide opportunities to strengthen electronic hospital management systems to enable health care providers to provide high quality care to their patients while harnessing the power of learning health systems.

In common with many developing countries, Kenya has experienced some success with several health informatics initiatives including the adoption of DHIS2 for centralised population data collection and OpenMRS for managing TB and HIV programmes in smaller clinics. This survey has highlighted important advancements in digitisation within Level 4–6 hospitals. However, it emerged from this survey that there is little harmonisation between the systems running in the CCC clinics and other departments within the hospital. We have shown that there was a dominance of digitisation within the registration, billing and outpatient department with little or no attempts to digitise any of the inpatient wards. We found only one system vendor who had attempted to provide an inpatient system as well as an administrative system. Where present, laboratory systems were often provided by a different vendor from the dominant system provider within the facility with some lab systems offering the capability to exchange data with other systems.

## Data Availability

The dataset supporting the quantitative findings of this article are presented in the tables contained in this publication. The qualitative data that support the findings of this study are available from the KEMRI-Wellcome Trust Research Programme, but restrictions apply to the availability of these data, according to the consenting process during the study. Those interested in the data can apply through the Data Governance Committee of the KEMRI-Wellcome Trust Research Programme.

## Appendix

**Table 4:** Coding overview for Hospital interviews

	Hospital interviews codes	Sample comments
Acquisition history	Financial Accountability: mention of financial accountability as reason to implement system	H13: Ok currently we depend on the user fee, because we collect the user fee from the patients, we also have the County government supporting us, and again we have partners who also contribute all this money is put in admnH4: Transactions done via the system aren’t reversible except by specific persons with such system privileges.
	Manage processes	
	Reason for acquisition: To manage clinical data Previous experience from others Previous system challenges Improved information (mentioned as a reason for system acquisition)	
	System selection and development process	
	Funding: initial funding and running costs, county funds, hospital funds	
	System initiator: Any person mentioned to have initiated system implementation	
Usability	Speed	
	Integration with other systems	H6: The system doesn’t allow changing of a radiology request during certain instances when it may be necessary. E.g. a clinician sends a patient for an x-ray for the left leg, when it really is the right leg that is injured and needs the x-ray done. H4: During power interruptions, any receipts that are printing or sent to print cannot be re-created
	User friendly: relating to the user interface and whether the users find it difficult to navigate	
	hanging/crashing	
	Work made easier	
	Decision support	
	Workflow/business logic	
	Govt requirements: Does the system meet government requirements? /Are any requirements from the Government that enable/hinder system use?	
	Computer literacy: relates to ability of users to use computers and software	
	Workarounds: Users using shortcuts to get system working	
	Time and Workload / reduce paperwork	
Report generation and data issues	Poor documentation	H13: For our consumption yes, like the financial report, commodity use reports we are able to know which drugs I need to stock, so we use a lot of the reports that we get from the program to make decisions
	Clinical data entry	
	Unavailable reports	
	Error reduction/improved accuracy	
	Report generation and access to reports: MOH reports, Local facility reports	
	Data confidentiality	
	Data quality: comments regarding ensuring data quality	
	missed data	
	Data extraction at facility	
	Data audits: mentions of ability to go back to data to counter-check issues	
	Data lookup and tracking	
	Inpatient data	
	Diagnosis and test availability	
Infrastructure issues	Hardware issues	H4: Power interruptions and fluctuations that slow down work. Power interruptions also cause problems with interchange of information with [system X] in the lab.
	Network issues	
	Electrical power interruptions	
	Theft/Equipment safety	
	Power fluctuations	
System support, acceptance and user training	Support by local staff: System user support provided by staff available at the hospital	H13: Most of the training is actually done by the IT team, but one of the guys you saw, a records officer is able to handle most of the clinical challenges and not just the IT personnel.
	Support by vendor, remote support	
	Response speed: relate to how fast or slow support is provided	
	Training: initial system training and ongoing training	
	Backup procedures: procedures in place in case system is not functioning	
	Procedure documentation	
	County IT support	
	Support prioritisation	
	System acceptance: persisting resistance, initial system resistance	
Departmental communication and system interoperability	System interoperability	H4: Connected to the CellTac FHG/CBC machine, allowing printing of reports and posting of results directly to the system.
	interdepartmental communication	

**Table 5:** Coding overview for Hospital interviews

	Vendor interviews codes	Sample comments
Data and reporting	Coded data	Vendor 5: the doctor can do the coding, and in most cases that is what happens, but in case where the module has not been bought, you know we sell it in models sometimes depending on resources availability and all that and I mean other things, so the health information people can still do it. We have a form as they collect the files the work has been done the people can still do the coding manually, but in our case, we prefer when the doctors are doing the coding themselves.
	Report generation	
	Unique identifiers	
	POC data entry	
	Retrospective data entry	
	Data transmission to DHIS2	
	Data export	
	Access to data or reports	
	Data quality	
Support to facilities	Remote support	Vendor 8: Ok it’s a bit unique, ok there are things which you can call over the phone and sort them outside and there is an issue of password, someone has forgotten a password you just direct them to a senior person who will go and rectify the password. Like if now it’s an issue about a report like now what I was talking about DHIS. Now that one has to be written formally, there is an email, it’s a kind of a letter that we respond to it we seek the way forward that why I am saying if something requires a meeting now we go and have a meeting with them
	In person support	
	Outsourced support	
	Documentation	
	Training	
	Support: simple/first level or advanced support	
	Maintenance contract	
	Hardware support	
	Local IT support	
	Issue tracking	
	Support prioritisation	
	Facility installations	
User Related	Positive attitude	Vendor 2: Maybe when they are not ready for training, you know sometimes you can go to a place where they have not dealt with computers and sometimes people find it very frightening to start using these things and all that, some of it can also be due to human factors, resistance to change, that inertia, so just the normal, normal things, when you are introducing a new thing,
	Negative attitude	
	Workload and time	
	Motivation to use system	
	User readiness	
System	Interoperability	Vendor 8: Those who are not very/ you know, those don’t have IT guys, they do external ones once in a week. Those who have IT guys, there is a day, there are some whom because of the sensitivity of the of the data and they sometimes collect a lot, they do backup straight, during the day they can do manual and wait for the one at night to be done automatically its only that they are limited in terms of the internet they have. If they had internet they wanted to be backing up back up outright in a cloud server somewhere. But you know when thy do the costing and all that sometimes they say that is a lot. So there are some facilities who have big data bases, they go around 500mb when it is zipped, and when it’s not zipped its around 3GB.
	Effect of system change	
	Setup process customisation and challenges	
	Architecture	
	Role based access	
	Backup - data dumps, location, redundancy, large files, costs, challenges, timing/frequency	
	Data protection - encryption	
	Backup - challenges	
	Modules: inpatient, important modules, new modules	
	Internet connectivity	
Legislation, Governance and National Programmes	MOH issues	Vendor 8: We can in fact the good thing about DHIS tool, we are using the same data base, we are using Postgres, they are Postgres we are Postgres, the only thing is that there has not been any agreement or the go ahead from the DHIS site for us to integrate
	Permission to access DHIS2	
	Integration with MOH requirements	
	Reduce resource wastage	
	County influence	

## Abbreviations

CCC: Comprehensive Care Clinic
CHRIO: County Health Records Information Officer
CPT4: Current Procedural Terminology
CWC: Child Welfare Clinic
DHIS2: District Health Information System Version 2
DICOM: Digital Imaging and communications in medicine
EHR: Electronic Health Records
EMR: Electronic Medical Records
HIV: Human Immunodeficiency Virus
HL7: Health Level 7
HMIS: Health Management Information System
HRIO: Health Records Information Officer
ICD10: The International Statistical Classification of Diseases and Related Health Problems 10th Revision
ICT: Information Communication Technology
IT: Information Technology
KEMRI: Kenya Medical Research Institute
LMIC: Low and Middle-Income countries
MOH: Ministry of Health
NHIF: National Health Insurance Fund
SNOMED-CT: Systematized nomenclature of medicine -Clinical Terms
